# Quality of Beverage Intake and Cardiometabolic and Kidney Outcomes: Insights From the STANISLAS Cohort

**DOI:** 10.3389/fnut.2021.738803

**Published:** 2022-01-07

**Authors:** Sandra Wagner, Thomas Merkling, Nicolas Girerd, Erwan Bozec, Laurie Van den Berghe, Axelle Hoge, Michèle Guillaume, Mehmet Kanbay, Céline Cakir-Kiefer, Simon N. Thornton, Jean-Marc Boivin, Ludovic Mercklé, Martine Laville, Patrick Rossignol, Julie-Anne Nazare

**Affiliations:** ^1^University of Lorraine, INSERM CIC 1433, Nancy CHRU, Inserm U1116, FCRIN INI-CRCT, Nancy, France; ^2^Centre de Recherche en Nutrition Humaine Rhône-Alpes, Univ-Lyon, CarMeN Laboratory, Université Claude Bernard Lyon1, Hospices Civils de Lyon, F-CRIN/FORCE Network, Lyon, France; ^3^Département des Sciences de la Santé Publique, Université de Liège, Liège, Belgium; ^4^Division of Nephrology, Department of Medicine, Koc University School of Medicine, Istanbul, Turkey; ^5^Université de Lorraine, INRAE, URAFPA, Nancy, France; ^6^Université de Lorraine, Inserm, DCAC, Nancy, France

**Keywords:** beverages, adult consumption, kidney function, cardiovascular health, metabolic health, Healthy Beverage Index

## Abstract

**Background and Aims:** Beverages are an important aspect of diet, and their quality can possibly affect health. The Healthy Beverage Index (HBI) has been developed to take into account these effects. This study aimed to highlight the relationships between health and beverage quality by assessing the association of the HBI and its components with kidney and cardiometabolic (CM) outcomes in an initially healthy population-based familial cohort.

**Methods:** This study included 1,271 participants from the STANISLAS cohort. The HBI, which includes 10 components of habitual beverage consumption, was calculated. Associations of the HBI and its components with estimated glomerular filtration rate (eGFR), albuminuria, hypertriglyceridemic waist (HTG waist), metabolic syndrome (MetS), carotid-femoral pulse wave velocity (cfPWV), carotid intima-media thickness (cIMT), and left ventricular mass (LV mass) were analyzed using multivariable linear or logistic regression models.

**Results:** The median HBI score was 89.7 (78.6–95) out of 100 points. While the overall HBI score was not significantly associated with any of the studied outcomes, individual HBI components were found differently associated with the outcomes. cfPWV and cIMT were lower in participants who did not meet the full-fat milk criteria (*p* = 0.03 and 0.001, respectively). In men, higher cfPWV was observed for the “low Fat milk” (*p* = 0.06) and “alcohol” (*p* = 0.03) non-adherence criteria. Odds of HTG waist were higher with the non-adherence to sugar-sweetened beverages criteria (*p* < 0.001). eGFR was marginally higher with non-adherence to the coffee/tea criteria (*p* = 0.047).

**Conclusions:** In this initially healthy population, HBI components were differently associated with kidney and cardiometabolic outcomes, despite a good overall HBI score. Our results highlight specific impacts of different beverage types and suggest that beverages could have an impact on kidney and cardiometabolic health.

## Introduction

Beverages are an important aspect of the diet as they provide the majority of water to the body (~80% of total water intake) (1, 2), nutrients, and calories (3). Studies have suggested that high water intake could be beneficial for the prevention of numerous diseases, such as chronic kidney disease (CKD) (1, 4) and diabetes (5). Other beverages can also have beneficial effects due to their nutritional content of bioactive molecules. For instance, drinking 3 or more cups of coffee per day has been shown to be associated with a reduced risk of chronic diseases, such as type 2 diabetes (6, 7) or cardiovascular (CV) disease (8). Being a coffee consumer may also be associated with a lower incidence of CKD (9). Conversely, sugar-sweetened beverages (SSBs) have deleterious effects on cardiometabolic health. SSBs are notably associated with metabolic syndrome (MetS), the onset of diabetes, CKD, and CV disease (10–12). Recently, industrial sweetened beverages have been shown to be associated with higher arterial stiffness (13). In a previous study, our group observed in young women that the dietary pattern characterized by the intake of sodas, among others, was positively associated with left ventricular mass (14). Regarding milk, its effect on health has been the subject of controversy in recent years due to the type of milk studied, its origin, its manner of consumption, and its contents, such as saturated fat, lactose, protein, and bifidobacteria (15, 16).

The European Food Safety Authority recommends a total water intake of 2 L/day for women and 2.5 L/day for men (2), albeit without any specific distinction between fluid sources. The French Programme National Nutrition Santé (PNNS) recommendations are to limit alcohol and SSBs, and to prefer water, ideally between 1 and 1.5 L/day of plain water (17). These guidelines do not provide clear information on other beverage types. Moreover, the commonly used dietary scores, such as HEI-2015, AHEI-2010, or MediSCORE, variably take into account only certain beverages, such as alcohol or milk (as part of dairy products) (18, 19). To overcome this limitation and to take into account the global quality of beverage intake, Duffey et al. (20) developed the Healthy Beverage Index (HBI). Similar to the dietary scores, the HBI serves as a measure of overall beverage quality by including 10 components of habitual beverage consumption.

Until now, few studies have investigated the association between HBI and health outcomes given the insufficiently accurate description of beverage intake in most of the studies for the calculation of this score. While higher HBI scores have been associated with more favorable lipid profiles and less hypertension risks (20), the relationship between overall beverage intake quality and kidney disease and the harder endpoint of cardiometabolic health in initially healthy adults are to date unknown.

The objective of this study was to assess the association of the HBI score and its components with kidney and cardiometabolic outcomes in the STANISLAS familial cohort study, which comprises 2 generations. This has been possible owing to a detailed and precise description of the diet, including the characteristics of all beverage intake, associated with an extensive CV phenotyping.

## Methods

### Study Population

**Figure 1:**
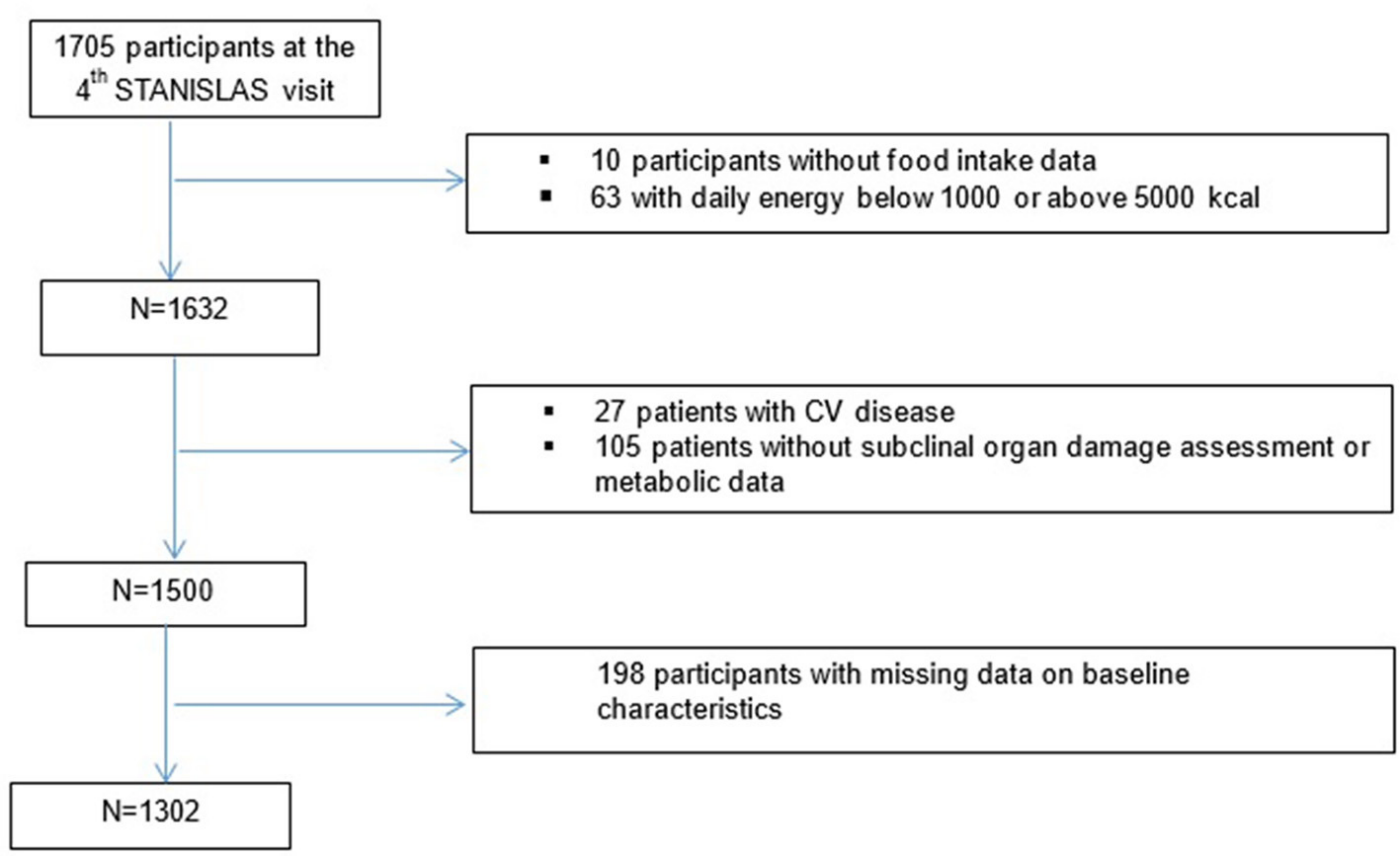
Flowchart of the study.

The STANISLAS cohort is a population-based study of 1,006 families each comprised of at least 2 parents and 2 children (4,295 participants) from the Lorraine region (Eastern France) recruited during 1993–1995 at the Center for Preventive Medicine. The participants were of French origin and free of acute or chronic disease. From 2011 to 2016, 1,705 participants underwent their fourth examination. The STANISLAS study has been described in detail elsewhere (21). The present study focused on the 1,695 participants who underwent their fourth examination and for whom data on food intake were available. After excluding 10 participants without food intake data, 63 with a daily energy intake either below 1,000 or above 5,000 kcal, 27 with cardiovascular (CV) disease (11 with myocardial infarction only, 12 with heart failure only, and 4 with both), 105 with at least one missing subclinical organ damage assessment (19 kidney, 82 CV, and 17 metabolic outcomes), and 198 with missing data regarding baseline characteristics, the present cross-sectional analysis included 1,302 participants (Figure 1).

The research protocols were all approved by the local Ethics Committee (Comité de Protection des Personnes Est III—Nancy—France), and all study participants gave written informed consent. The informed written consent was previously approved by the local Ethics Committee.

### Data Collection

#### Dietary Assessment

Dietary intake was assessed using a validated food frequency questionnaire (FFQ) (22). The participants reported their consumption frequency and portion size of 133 food and beverage items over the previous 3 months. Consumption frequency was reported using 6 levels in the questionnaire, ranging from “never or rarely” to “2 times or more a day.” The portion size of each food or beverage item was estimated using standard serving sizes and food models. Daily nutrient intakes were calculated in grams per day by multiplying the consumption frequency of each item by the nutrient content of selected portions.

Nutritional data were extracted from the French food composition database established by the French Data Center on Food Quality (Ciqual, last updated in 2013).

#### Healthy Beverage Index

The HBI is a score assessing overall beverage intake quality in relation to total daily energy and fluid needs, against standards set by the Dietary Guidelines for Americans and the Beverage Guidance Panel (20). The recommendations were converted to fluid needs as a percentage of total fluid requirements. The HBI score is the sum of 10 sub-component scores: 8 beverage categories, total beverage energy, and fluid consumption (Table 1). The standard fluid requirement of 1 ml/kcal consumed was used to determine total fluid needs. The HBI score ranges from 0 to 100, with higher scores indicating greater adherence to beverage recommendations and guidelines.

**Table 1:** Healthy Beverage Index (HBI) components developed by Duffey et al. (20).

**Component**	**Description**	**Points**
Water	Water comprises at least 20% of fluid requirements	15
	No water consumption	0
	Water <20% of fluid requirements	Proportional
Coffee and tea	Unsweetened coffee and tea comprise 0–40% of fluid requirements	5
Low-fat milk	<1.5%, fat-free, and/or soy milk comprises 0–16% of fluid requirements	5
Diet drinks	Artificially sweetened beverages comprise 0–16% of fluid requirements	5
100% fruit juice	100% fruit juice comprises 0–8% of fluid requirements	5
Alcohol	Between 0–1 drinks for women, 0–2 drinks for men	5
Full-fat milk	0% of fluid requirements come from 2% fat or full-fat milk	5
Sugar-sweetened beverages	Sugar-sweetened beverages are 0–8% of fluid requirements	15
Total beverage energy	Energy from beverages <10% of total energy	20
	Energy from beverages ≥ 10% but <15% of total energy	Proportional
	Energy from beverages ≥ 15% of total energy	0
Met fluid requirements	Amount of beverages (mL) consumed was greater than or equal to fluid requirements	20
	Amount of beverages (mL) consumed was less than fluid requirements	Proportional

### Cardiometabolic Outcomes

#### Hypertriglyceridemic Waist

Hypertriglyceridemic (HTG) waist was defined as follows: waist circumference (WC) > 88 cm and triglycerides > 1.50 g/L for women; WC > 102 cm and triglycerides > 1.50 g/L for men (23).

#### Metabolic Syndrome

Metabolic syndrome was defined according to the National Cholesterol Education Program ATP3 (24) as the presence of 3 or more of the following components: increased WC (> 102 cm for men and > 88 cm for women); increased levels of triglycerides (≥ 1.5 g/L) or treated with lipid-lowering drugs; reduced high-density lipoprotein cholesterol (HDL-C) (<0.4 g/L for men, and <0.5 g/L for women); increased office systolic blood pressure (SBP, ≥ 130 mm Hg), or increased diastolic blood pressure (DBP, ≥ 85 mm Hg), or treated with antihypertensive drugs; or elevated glucose (≥ 1.10 g/L) or treated with glucose-lowering drugs.

#### Carotid-Femoral Pulse Wave Velocity

Carotid-femoral pulse wave velocity (cfPWV) was measured using the Complior device (Alam Medical, France) in a quiet room after at least 10 min of rest in a supine position according to the recommendations of the European Network for the Non-invasive Investigation of Large Arteries (21, 25, 26). Two sensors were placed simultaneously on the carotid artery and femoral artery. Two measurements were made, with cfPWV calculated as their mean. If the 2 measurements differed by >0.5 m/s, a third measurement was made, and the cfPWV was then calculated as the median of the 3 measurements. The onboard foot-to-foot algorithm based on the second-derivative waveforms was used to determine the transit time. The carotid-to-femoral, carotid-to-sternal-notch, and sternal-notch-to-carotid distances were measured with a measuring tape. The distance used for cfPWV calculation was 0.8 times the direct carotid-femoral distance, with cfPWV calculated as distance divided by the transit time.

#### Carotid Intima-Media Thickness

Carotid intima-media thickness (cIMT) measurements were routinely performed by high-resolution echocardiographic tracking as previously described (27). The non-invasive investigations were performed in a controlled environment at 22 ± 1°C after 10 min of rest in a supine position. Carotid diameter, carotid distention, and cIMT were measured for the right common carotid artery. Four measurements were carried out per patient. Examinations were performed with a wall tracking system (ESOATE, Maastricht, The Netherlands) and/or the ART.LAB (ESAOTE) in immediate succession. Both inter-device reproducibility and measurement agreement were excellent (28).

#### Left Ventricular Mass

Echocardiographic examinations were performed in the left lateral decubitus position by an experienced echocardiographer using a commercially available standard ultrasound scanner (Vivid E9, General Electric Medical Systems, Horten, Norway) with a 2.5-MHz phased-array transducer (M5S). The echocardiography/Doppler examinations included exhaustive examinations in parasternal long- and short-axis views and in the standard apical views (29, 30). All acquired images and media were stored on a secured network server as digital videos with unique identification numbers, and subsequently analyzed on a dedicated workstation (EchoPAC PC, version 110.1.0, GE Healthcare, IL, USA). Septal wall thickness, posterior wall thickness, and left ventricular (LV) internal diastolic diameter were measured from the parasternal 2-dimensional long-axis view. These measurements were subsequently used in the cube-function formula of the American Society of Echocardiography guidelines to calculate LV mass (31), which was then indexed for height to the 2.7 power (32).

#### Kidney Outcomes

Kidney function was assessed by calculation of the estimated glomerular filtration rate (eGFR) using the CKD-EPI equation (33).

Urine albumin-to-creatinine ratio (ACR) was calculated from a spot urine sample. ACR was dichotomized according to class (<90th percentile).

#### Covariates

A self-reported questionnaire was used to collect the demographic and socioeconomic information, such as age, sex, education level (categorized into low, intermediate, or high), and smoking status (categorized into yes or no), as well as information relative to disease history and treatment (21). Physical activity was assessed using the International Physical Activity Questionnaire (14).

Anthropometric measurements, such as weight, height, and WC were performed during clinical examination. Blood samples were collected, and the serum concentrations of the following biomarkers were measured, i.e., fasting glucose, HDL-C, and triglycerides (21). Office blood pressure was also measured (21), as well as 24-h ambulatory blood pressure (27, 34). In brief, participants underwent 24-h recording of the ambulatory blood pressure using the Spacelabs 90207 ambulatory monitor (Spacelabs Medical, WA, USA), with the monitoring cuff placed around the non-dominant arm. The blood pressure system was programmed to perform measurements every 15 min from 6 A.M. to 10 P.M. and every 30 min from 10 P.M. to 6 A.M.

### Statistical Analysis

Participant characteristics were first described according to total water intake according to HBI quartiles. Differences between the groups were assessed using Fisher's exact test or the chi-square test for categorical variables, and the Kruskal–Wallis non-parametric test for continuous variables.

A graphical representation of HBI component scores was also preformed for the overall population and by generation (Generation 1: ≥ 50 years and Generation 2: <50 years) and sex using a radar chart. The association between HBI or each of its components with each kidney and cardiometabolic outcome was then investigated. Of note, HBI component 4 (diet drinks) alone was not considered since only 22 participants (<2%) had a score different from the majority. Due to the family structure of the STANISLAS cohort, a family random intercept was added in each model to account for the non-independency of members of the same family. For continuous outcomes, linear mixed models with the Kenward-Roger correction for degrees of freedom were used to calculate *p*-values from conditional *F*-tests and 95% profile *CI*s of estimates, using the *lmerTest* package (35). For binary outcomes, the binomial generalized linear mixed models (with a logit link) were used using the *glmmTMB* package (36) and *p*-values were calculated using likelihood-ratio tests. In addition, to ascertain whether the associations between HBI or its components and the studied outcomes were dependent on generation or sex, data were tested for 2-way interactions between HBI or its components and generation or sex (except for binary outcomes, due to the very small number of events). The interaction between HBI and total energy intake was also tested, although the latter was not significant (all *p* > 0.11). Covariates were selected using a two-step process: (1) univariable multinomial logistic regressions were run with HBI in quartiles as the response variable and variables with *p* < 0.2 were retained, and (2) a multiple multinomial logistic regression was run with all variables selected during the first step from which variables with *p* < 0.05 were retained as covariates. The selected covariates were sex, smoking status, and energy intake, the models were further adjusted for age (standardized within sex and generation), generation, income, BMI (body mass index) class, and physical activity. For cardiometabolic outcomes, the models were further adjusted for DBP. To improve model convergence, energy intake and physical activity were standardized. When considering an HBI component as exposure variable, the results were adjusted for the other components, except component 1 (water) due to its collinearity with component 10 (beverage volume). This meant that when considering component 1, the latter was not adjusted for component 10. Model residual checks led to the log-transformation of cIMT and pulse-wave velocity. To facilitate interpretation, beta coefficient and 95% *CI* were exponentiated for these models.

## Results

### Characteristics of the Study Population

**Figure 2:**
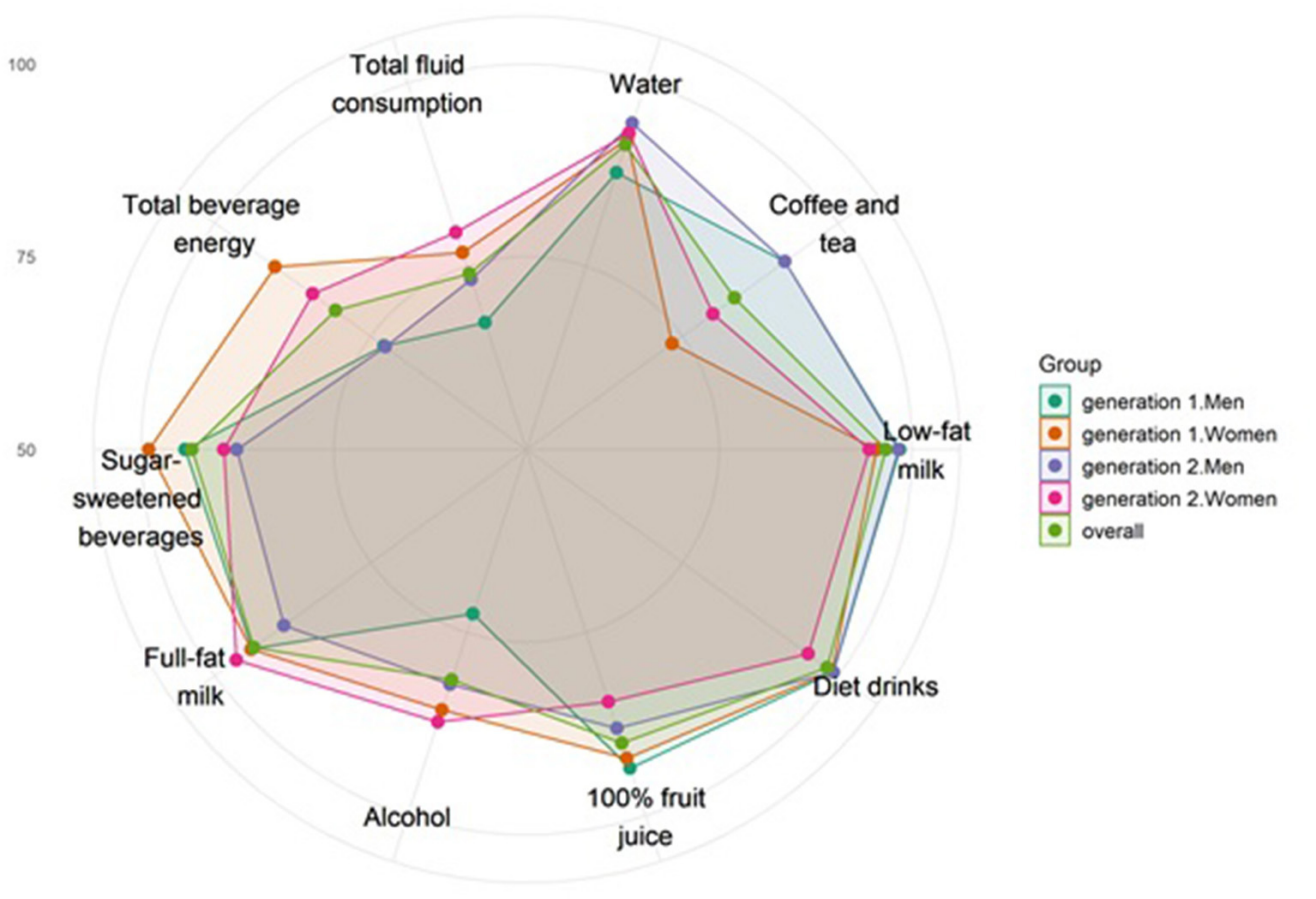
Graphical representation of mean relative Healthy Beverage Index (HBI) component scores for the overall population and according to generation and sex. The scale is relative to the maximum score of each HBI component. For example, a score of 100% represents the case when all participants reached the maximum score, whereas a score of 50% represents the case when the average score was half of a maximum score. The center of the figure represents a mean of 50% of the maximum score, the smallest circle represents 75%, and the second circle represents 100%. Generation 1, oldest generation; Generation 2, youngest generation.

The median (Q1-Q3) HBI score was 89.7 (78.4–95). Details of all beverage consumptions are presented in Supplementary Table 1. The HBI score was higher (*p* < 0.001) in women in both generations [91.8 (85.8–95) for Generation 1 (≥ 50 years) and 90.3 (81.8–95) for Generation 2 (<50 years)] compared with men [86.3 (74.8–93.3) for Generation 1, 88.1 (75.0–93.8) for Generation 2]. Differences were observed for sex and generation among the different HBI components. For both generations, women had lower scores than men for “coffee and tea,” indicating that they drank more coffee or tea than recommended. Generation 1 men had the lowest scores for “alcohol,” “total beverage energy,” and “total fluid consumption” (Figure 2).

Participants in the highest HBI quartile (>95) were more likely to be women and non-smokers, have higher incomes, lower energy intake, and blood pressure (Table 2).

**Table 2:** Description of the study population as a whole and according to HBI quartiles.

	**Overall**	**≤78**	**79–89**	**90–95**	**>95**	***p***
N	1,302	313	335	325	329	
Age (yrs)	56.0 [34.2, 60.0]	56.0 [34.0, 61.0]	56.0 [35.0, 60.0]	56.0 [35.0, 60.0]	55.0 [35.0, 60.0]	0.905
Women (%)	683 (52)	36	52	58	64	<0.001
Generation 2 (%)	523 (40)	42	39	39	41	0.816
Smoking status (%)						<0.001
Non-smoker	624 (48)	37	51	52	50	
Previous smoker	416 (32)	33	29	32	34	
Current smoker	262 (20)	30	20	15	15	
Education level (%)						0.381
Bachelor's degree	536 (41)	44	43	36	41	
2-y university degree	439 (34)	30	34	36	34	
> 2-y university degree	327 (25)	26	23	27	24	
Income (%)						0.098
<2,249€/month	409 (31)	36	30	30	29	
2,250–2,999€/month or no answer	295 (23)	25	24	19	22	
>3,000€/month	598 (46)	39	46	50	48	
BMI (kg/m^2^)	25.0 [22.5, 28.2]	24.8 [22.4, 27.8]	25.2 [22.5, 28.1]	25.1 [22.7, 28.5]	24.8 [22.4, 28.1]	0.752
BMI class (%)						0.738
Normal	657 (50)	52	49	49	52	
Overweight	433 (33)	33	36	34	31	
Obese	212 (16)	15	15	18	17	
Waist circumference (cm)	89.2 (13.4)	90.6 (13.2)	89.4 (13.2)	88.9 (13.5)	88.1 (13.6)	0.119
Waist-hip ratio	0.9 [0.9, 1.0]	0.9 [0.9, 1.0]	0.9 [0.8, 1.0]	0.9 [0.8, 1.0]	0.9 [0.8, 1.0]	<0.001
Energy intake (kcal/d)	2359.7 (806.1)	2598.9 (869.3)	2620.2 (875.7)	2306.5 (670.2)	1919.4 (559.4)	<0.001
Physical activity (MET/min)	1875.0 [741.3, 4243.0]	1965.3 [678.0, 4746.0]	1920.0 [849.0, 4601.1]	1752.0 [636.0, 3775.8]	1801.8 [801.0, 4158.0]	0.260
Diabetes (%)	60 (5)	4	5	4	5	0.697
Fasting blood glucose (g/L)	0.9 [0.8, 0.9]	0.9 [0.8, 1.0]	0.9 [0.8, 0.9]	0.9 [0.8, 0.9]	0.9 [0.8, 0.9]	0.505
Glycated hemoglobin (%)	5.6 [5.3, 5.8]	5.5 [5.3, 5.8]	5.6 [5.4, 5.8]	5.6 [5.3, 5.8]	5.6 [5.3, 5.8]	0.266
Use of antidiabetic drugs (%)	45 (3)	3	3	3	4	0.851
Hypertriglyceridemia (%)	344 (26)	29	26	23	27	0.336
Triglycerides (g/L)	0.9 [0.7, 1.3]	1.0 [0.7, 1.3]	0.9 [0.7, 1.2]	0.9 [0.7, 1.2]	0.9 [0.7, 1.2]	0.214
Use of lipid-lowering drugs (%)	192 (15)	15	16	12	17	0.281
LDL cholesterol (g/L)	1.3 (0.3)	1.3 (0.3)	1.3 (0.3)	1.4 (0.3)	1.3 (0.3)	0.249
HDL cholesterol (g/L)	0.6 (0.1)	0.6 (0.1)	0.6 (0.1)	0.6 (0.1)	0.6 (0.2)	0.052
Hypertension (%)	518 (40)	44	40	39	37	0.250
24 h DBP (mmHg)	74.3 (7.2)	75.7 (7.5)	74.2 (7.2)	74.1 (7.0)	73.3 (6.8)	<0.001
24 h SBP (mmHg)	120.1 (10.1)	122.5 (10.6)	120.3 (9.9)	119.7 (9.6)	118.0 (9.8)	<0.001
Use of antihypertensive drugs (%)	246 (19)	19	20	18	19	0.911
Hypertriglyceridemic waist (%)	95 (7)	8	8	6	6	0.687
Metabolic syndrome (%)	303 (23)	24	24	21	24	0.627
Intima media thickness (μm)	628.8 (142.3)	638.2 (145.1)	628.2 (136.3)	623.3 (139.1)	625.9 (148.8)	0.585
Pulse wave velocity (m/s)	8.2 [7.3, 9.3]	8.3 [7.4, 9.6]	8.2 [7.4, 9.3]	8.2 [7.3, 9.4]	8.1 [7.3, 9.1]	0.266
Left ventricular mass (g/height^2^ 0.7)	73.3 [62.3, 86.3]	75.0 [63.6, 88.4]	74.8 [64.2, 85.4]	72.8 [61.3, 84.7]	71.5 [60.6, 85.7]	0.092
eGFR (ml/min/1.73 m^2^)	32.4 [27.4, 39.3]	32.4 [28.0, 39.7]	32.8 [27.4, 39.4]	32.8 [27.4, 39.2]	31.8 [27.1, 39.0]	0.724
Elevated urine albumin/creatinine ratio (%)	96.3 [87.1, 107.0]	97.3 [87.6, 109.0]	95.8 [86.4, 106.0]	96.1 [86.7, 108.6]	96.8 [87.2, 107.0]	0.537

*Continuous variables are expressed as mean ± SD or median (Q1–Q3) as appropriate. Hypertension is defined as increased blood pressure (130/80) and/or declared hypertension and/or use of at least 1 antihypertensive drug; diabetes mellitus is defined by high fasting glucose (>1.26 g/L) and/or declared diabetes mellitus and/or use of at least 1 antidiabetic drug. Generation 2 correspond to the youngest individuals, related to generation 1*.

*BMI, body mass index; DBP, diastolic blood pressure; HDL, plasma high-density cholesterol; LDL, plasma low-density cholesterol; MET, metabolic equivalent task; SBP, systolic blood pressure*.

### Association Between Overall HBI and Kidney or Cardiometabolic Outcomes

There was no strong evidence for an association between any of the studied outcomes and HBI (in quartiles or continuous, Table 3).

**Table 3:** Association of HBI with kidney and cardiometabolic outcomes.

	**Hypertriglyceridemic waist OR (95% CI)**	**Metabolic syndrome OR (95% CI)**	**PWV exp(beta) (95% CI)**	**cIMT exp(beta) (95% CI)**	**LV mass beta (95% CI)**	**eGFR beta (95% CI)**	**ACR OR (95% CI)**
HBI quartiles	*p =* 0.67	*p =* 0.48	*p =* 0.72	*p =* 0.64	*p =* 0.98	*p =* 0.85	*p =* 0.50
≤ 78	1.12 [0.15–8.41]	0.97 [0.61–1.55]	1 [0.98; 1.03]	1 [0.98; 1.03]	−0.2 [−1.48; 1.09]	−0.04 [−1.81; 1.74]	0.98 [0.56–1.7]
79–89	1.92 [0.3–12.07]	1.02 [0.65–1.61]	1.01 [0.99; 1.04]	0.99 [0.96; 1.02]	−0.17 [−1.41; 1.08]	−0.68 [−2.39; 1.04]	0.96 [0.56–1.66]
90–95	0.64 [0.12–3.42]	0.75 [0.48–1.17]	1.01 [0.99; 1.03]	0.99 [0.96; 1.01]	0.05 [−1.17; 1.26]	−0.2 [−1.87; 1.47]	0.68 [0.39–1.19]
> 95	Ref	Ref	Ref	Ref	Ref	Ref	Ref
Continuous HBI	*p =* 0.53	*p =* 0.93	*p =* 0.96	*p =* 0.64	*p =* 0.51	*p =* 0.92	*p =* 0.69
slope	1.21 [0.67–2.21]	0.99 [0.87–1.14]	1 [0.99; 1.01]	1 [0.99; 1.01]	0.12 [−0.24; 0.49]	−0.02 [−0.53; 0.48]	0.97 [0.82–1.14]

*Binary outcomes were adjusted for sex, age, generation, smoking status, energy intake, BMI class, income, beverage volume, and total beverage energy. Continuous outcomes were adjusted for sex, age, generation, smoking status, energy intake, BMI class, income, and physical activity (+ DBP for PWV, cIMT, and LV mass)*.

*PWV, pulse wave velocity; cIMT, carotid intima-media thickness; LV mass, left ventricular mass, indexed for height to the 2.7 power; eGFR, estimated glomerular filtration rate; ACR, albumin to creatinine ratio*.

### Association Between HBI Components and Cardiometabolic Outcomes

When looking at each HBI component individually, the odds of HTG waist were higher among participants who did not meet the “sugar-sweetened beverages” (0–8% of fluid requirements) criteria (*p* = 0.009; Table 4).

**Table 4:** Association between each component of the HBI and kidney and cardiometabolic outcomes (total *N* = 1,302).

**HBI component**		**HTG Waist OR (95% CI)**	**Metabolic syndrome OR (95% CI)**	**cfPWV exp(beta) (95% CI)**	**cIMT exp(beta) (95% CI)**	**LV mass beta (95% CI)**	**eGFR beta (95% CI)**	**ACR OR (95% CI)**
Water	score <15 (*N* = 235) vs. 15	0.44 [0.06–3.07]	1.05 [0.67–1.65]	1 [0.98; 1.03]	1.02 [0.99; 1.04]	−0.73 [−1.86; 0.4]	0.46 [−1.1; 2.03]	1.13 [0.64–2]
	*P*-value	0.40	0.83	0.67	0.19	0.21	0.57	0.68
Coffee and tea	score 0 (*N* = 211) vs. 5	0.49 [0.07–3.32]	0.66 [0.41–1.08]	1.01 [0.98;1.03]	Generation 1: 1.01 [0.98; 1.05] Generation 2: 2.02 [–0.67; 4.71]	−0.17 [−1.51; 1.16]	1.76 [–0.08; 3.6]	0.76 [0.41–1.38]
	*P*-value	0.45	0.093	0.62	0.084	0.80	0.063	0.35
Low-fat milk	score 0 (*N* = 42) vs. 5	0.31 [0–28.1]	1.34 [0.55–3.26]	Men: 1.09 [0.99; 1.2] Women: 0.99 [0.94; 1.04]	1.04 [0.98; 1.1]	−0.75 [-3.28;1.78]	Generation 1: 3.76 [–0.87; 8.39] Generation 2: –6.47 [–11.31; –1.63]	2.29 [0.89–5.91]
	*P*-value	0.58	0.52	0.071	0.17	0.56	0.002	0.11
100% fruit juice	score 0 (*N* = 126) vs. 5	0.82 [0.09–7.81]	0.8 [0.47–1.38]	0.99 [0.96; 1.02]	1 [0.96; 1.03]	−1.15 [−2.67; 0.36]	0.9 [−1.19; 2.99]	1.02 [0.52–2.01]
	*P*-value	0.86	0.42	0.57	0.78	0.14	0.40	0.96
Alcohol	score 0 (*N* = 237) vs. 5	0.14 [0.01–1.88]	1.03 [0.65–1.62]	Men: 1.01 [0.98; 1.05] Women: 0.99 [0.94; 1.04]	0.99 [0.96; 1.02]	0.82 [−0.57; 2.21]	1.8 [–0.11; 3.71]	1.43 [0.78–2.61]
	*P*-value	0.1	0.91	0.047	0.56	0.25	0.067	0.25
Full-fat milk	Score 0 (*N* = 80) vs. 5	3.61 [0.37–35.06]	0.66 [0.34–1.26]	0.96 [0.92; 0.99]	0.94 [0.9; 0.97]	−0.37 [−2.16; 1.42]	1.59 [−0.89; 4.07]	0.8 [0.34–1.9]
	*P*-value	0.27	0.19	0.01	0.001	0.69	0.21	0.60
Sugar-sweetened beverages	Score 0 (*N* = 83) vs. 15	26.13 [1.81–377.3]	1.12 [0.59–2.13]	0.98 [0.95; 1.02]	1.01 [0.97; 1.05]	1.73 [−0.29; 3.75]	−1.03 [−3.82; 1.75]	1.61 [0.73–3.53]
	*P*-value	0.009	0.72	0.35	0.68	0.1	0.47	0.25
Total beverage energy	score <20 (*N* = 354) vs. 20	1 [0.23–4.32]	1.08 [0.77–1.51]	1.01 [0.98; 1.03]	1.02 [1; 1.05]	−0.41 [−1.68; 0.86]	0.77 [−0.97; 2.52]	0.84 [0.54–1.32]
	*P*-value	0.99	0.66	0.53	0.082	0.53	0.39	0.46
Beverage volume	Score ≤ 12.6 (*N* = 425) vs. > 18.7	0.16 [0.03–1]	1.11 [0.71–1.71]	1.01 [0.98; 1.04]	1.01 [0.98; 1.04]	–1.5 [–2.94; –0.07]	−0.17 [−2.16; 1.82]	1.36 [0.79–2.37]
	Score 12.6–18.7 (*N* = 428) vs. > 18.7	0.21 [0.04–1.11]	1.19 [0.81–1.75]	1.02 [1;1.04]	1 [0.98; 1.03]	–0.06 [–1.23; 1.11]	0.22 [−1.4; 1.83]	1.05 [0.63–1.74]
	*P*-value	0.087	0.68	0.22	0.89	0.039	0.88	0.469

*Binary outcomes were, adjusted for sex, age, generation, smoking status, energy intake, BMI class, income, beverage volume, and total beverage energy. Continuous outcomes were adjusted for sex, age, generation, smoking status, energy intake, BMI class, income, and physical activity (+ DBP for PWV, cIMT, and LV mass), and for the other HBI components, except “water.” When “water” was the exposure variable, the latter was not adjusted for “beverage volume” due to colinearity issues*.

*HTG Waist, hypertriglyceridemic waist; cfPWV, carotid-femoral pulse wave velocity; cIMT, carotid intima-media thickness; LV mass, left ventricular mass; eGFR, estimated glomerular filtration rate; ACR, albumin to creatinine ratio*.

The “beverage volume” score was associated with LV mass, with lower LV mass for participants who did not meet the criteria (−1.5 [−2.94; −0.07], Table 4). Moreover, participants who did not meet the “full-fat milk” criteria (0% of fluid requirements) had lower cfPWV and cIMT (0.96 [0.92; 0.99] and 0.94 [0.90; 0.97], respectively, Table 4). Sex-specific effects were observed for the association of “low fat milk” / “alcohol” with cfPWV. Men who did not meet the criteria for “low-fat milk” and “alcohol” (0–2 drinks) had higher cfPWV, whereas the reverse was true for women (0–1 drinks) (Table 4).

### Association Between HBI Components and Kidney Outcomes

The association of “low-fat milk” with eGFR was driven by generation. Generation 2 participants who did not meet the criteria for “low-fat milk” (0–16% of fluid requirement) had a lower eGFR while the reverse was true for Generation 1 participants, although to a lesser extent (Generation 1 (≥ 50 years): 3.76 [−0.87; 8.39]; Generation 2 (<50 years): −6.47 [−11.31; −1.63], Table 4). Participants who did not meet the criteria for “coffee and tea” (0–40% of fluid requirements) had a trend for higher eGFR (1.76 [−0.08; 3.6], Table 4). No associations were observed between HBI components and albuminuria.

## Discussion

In this cohort of initially healthy individuals, the HBI score was relatively high. The HBI score, reflecting the quality of overall beverage intake, was not significantly associated with any of the studied outcomes. However, when focusing on the individual HBI components, the latter were differently and independently associated with the cardiometabolic and kidney outcomes.

### Overall HBI Score and Outcomes

In the NHANES study, higher HBI was associated with more favorable lipid profiles, lower hypertension risk in men, and lower CRP, but not with MetS (20). Similarly, in the present study, no associations were found between HBI score and either MetS or subclinical organ damage. The HBI score was relatively high [89.7 (78.6–95)] in our cohort compared with that of the NHANES study (63 ± 16) (20). The differences in HBI scores between the two cohorts could be due to two factors. First, our cohort was comprised of initially healthy individuals, along with a relatively homogenous global consumption, thereby limiting the observation of an association with the studied outcomes. Second, culture can influence eating behaviors including beverage intake (37, 38). Although water is the major contributor of beverage consumption in the United States and in France (39–41), the United States is nonetheless characterized by higher consumption of SSBs and milk (37, 42). The total fluid intake was seemingly higher in the United States compared with the present study and other studies in France. It is noteworthy to emphasize that both water and fluid intake were higher in the present study compared with other French studies (39, 43–45). Having different drinking patterns across countries can lead to different scores, thus, the associated risks may not be the same between studies. Of note, the HBI components herein were associated with different outcomes. One may speculate whether compensation between the consumed beverages may counterbalance the overall score and result in the absence of association.

### HBI Components and Cardiometabolic Outcomes

Sugar-sweetened beverages are the largest source of added sugar in the diet and have consequently drawn much attention related to their etiological role in relation to obesity, type 2 diabetes, and CV risk (11, 46, 47). These associations may be partly mediated by adiposity (48). In the European PREDIMED study, higher SSB consumption (> 5 servings/week) was positively associated with MetS incidence (49). Nonetheless, the association between SSB and the presence of MetS remains less consistent (11), whereby associations are seemingly stronger when MetS components are considered individually. This discrepancy has been largely discussed due to the still much-debated clinical utility, consensus assessment, and heterogeneity of MetS.

In the present study, no association was found with MetS. Interestingly, there was a significant association between HTG waist and SSB consumption, although this must be considered cautiously in view of the low number of cases. This observation is in line with previous systematic review data demonstrating positive associations for blood pressure, triglycerides, low-density lipoprotein (LDL) cholesterol, and blood glucose, with an inverse association observed for high-density lipoprotein (HDL) cholesterol (50), and abdominal adiposity (11, 48).

With regard to CV outcomes, high SSB intake has been associated with a higher risk of stroke and myocardial infarction (50, 51). We did not find an association with subclinical vascular damage, i.e., with either IMT, in accordance with a previous study in middle-aged US women (52), or cfPWV, whereas another study conversely reported an association between increased intake in fructose derived from industrial sweetened beverages (> 1 drink/day) and higher cfPWV (13).

Having an adequate fluid intake is essential to avoid dehydration and the subsequent development of health issues or chronic diseases (53). In addition, hydration can have an influence on heart function and hemodynamic response. Indeed, blood volume is regulated by matching total water input and output (54). In the present study, drinking fewer beverages than the recommended fluid requirement was associated with lower LV mass. Nonetheless, this criterion was calculated based on consuming 1 ml liquid for each 1 kcal food consumed, which may be approximative, since not taking into account other lifestyle and personal determinants.

Regarding alcohol, men who did not meet this criterion (0–2 drinks/day) had higher cfPWV. This result is consistent with other studies showing that alcohol consumption is associated with higher cfPWV (55). Interestingly, in a study involving the same cohort, our group previously identified an alcohol pattern in Generation 1 men, and a fast-food and alcohol pattern in Generation 2 men whereby the former was positively associated with cfPWV, but not the latter (14). The present study further revealed that when focusing on beverages only, regardless of the generation, men who did not meet the criteria of alcohol intake had higher cfPWV. The fact that no association was observed for women could be the result of men adhering less to the criteria than women, especially Generation 1 men. Furthermore, the impact of alcohol on CV damage may differ between women and men, in relation to their specific cardiometabolic profiles (56). However, only a whole alcohol category was used in the HBI, i.e., the type of alcohol was not specified, whereas the type of alcoholic beverages may differentially affect the vascular system (57).

The effect of milk on health has been controversial in recent years due to its nature and content in saturated fat, although the presence of some peptides from whey proteins and caseins may have several biological activities (hypocholesterolemic, antihypertensive, antithrombotic, or antioxidant) that have potential beneficial effects on cardiometabolic risks (58). In a recent meta-analysis, milk intake was found to be associated with a reduced risk of hypertension, but not CVD (16). Moreover, the PURE study showed that the consumption of dairy products was associated with a lower risk of mortality and major events related to CV disease (59). Even if low-fat dairy products have become popular in response to the needs of consumers for reduced fat in food products (60), both low-fat dairy products and whole milk have been associated with lower risks of hypertension (60, 61) and better CV health (62). Nevertheless, the HBI score set at 0% of fluid requirements is based on 2% or full-fat milk. In the present study, we observed that drinking full-fat milk was negatively associated with cfPWV and cIMT. In contrast, drinking low-fat milk above 0–16% of fluid requirements was positively, although marginally, associated with PWV in men only.

### HBI Components and Kidney Outcomes

Drinking low-fat milk above the 0–16% of fluid requirements was associated herein with a lower eGFR in Generation 2 only. Another study found no association between daily consumption of milk, milk products, or low-fat dairy and annual eGFR decline whereas, in a subgroup of participants with mildly decreased eGFR, daily consumption of ≥2 servings of milk and milk products or low-fat dairy was associated with less annual decline in eGFR (63). Their findings are thus in contradiction with the current study. Further studies are therefore needed to better understand this particular aspect. An important feature to take into consideration is the normal kidney function observed in the present cohort.

Coffee and tea contain a mixture of components, notably polyphenols, featuring antioxidant and anti-inflammatory properties. Tea and coffee intake have been associated with multiple health-related effects, notably a decreased prevalence of certain diseases, such as cancer or metabolic diseases (6). However, few studies have investigated the association between coffee intake and CKD prevalence, even less for tea, the results of which are controversial. Of two recent meta-analyses, one found an association between coffee intake and a lower risk of incident CKD (9) while the other did not find any significant association (6, 64). The present study may suggest that drinking coffee and tea beyond 0–40% fluid requirements is associated with higher eGFR. While this criterion is binary, the protective effects of coffee nonetheless appear to be dose-dependent (65). Given that the STANISLAS population displayed a normal eGFR on average, we were unable to focus on CKD *per se*, although it can be inferred from our results that participants who met the coffee and tea criteria may exhibit a better preserved kidney function.

Overall, the HBI could help in implementing the specific guidelines on all beverage types and amounts for the general population, completing the existing guideline on water and alcohol (2, 17) intake and the extended guideline on SSBs (66).

The strengths of this study are multiple. First, the analyses were based on a large general and initially healthy population-based cohort. Second, our cohort allowed investigating the effects of generation and sex. Third, this study is based on quality data owing to the availability of comprehensive and detailed information relative to diet, kidney function, and extensive CV phenotyping.

However, certain limitations of this study should be acknowledged. First, the results are based on cross-sectional data and thus causality cannot be implied due to the study's observational nature. Second, the HBI was designed based on an American population and recommendations. Nonetheless, the guidelines in terms of beverages are partial.

## Conclusion

In this initially healthy population, the overall HBI score was excellent and was not associated with any kidney or cardiometabolic outcomes in either generation, men or women. When focusing on the HBI components individually, the latter were differently associated with the studied outcomes. Our results suggest the potential health benefits of achieving specific beverage intake guidelines with regard to kidney and cardiometabolic health. Accordingly, the present findings highlight specific impacts of different beverage types and suggest a need for specific guidelines regarding the types/amounts of beverages to consume, while also taking into consideration the total volume of beverages.

## Data Availability Statement

The raw data supporting the conclusions of this article will be made available by the authors upon reasonable request.

## Ethics Statement

The studies involving human participants were reviewed and approved by Comité de Protection des Personnes Est III—Nancy—France. The participants provided their written informed consent to participate in this study.

## Author Contributions

PR, NG, and J-MB designed the fourth visit of the STANISLAS cohort. Food data were verified and corrected by LV, SW, J-AN, and AH. LM performed the data management of the data. EB and NG supervised the cardiovascular (arterial stiffness and thickness and echocardiography, respectively) assessments. SW, J-AN, J-MB, NG, and PR designed the present research. TM performed the statistical analysis. SW and J-AN drafted the manuscript. All authors were involved in the interpretation of the results and the critical review of the manuscript and approved the manuscript.

## Funding

The fourth examination of the STANISLAS study was sponsored by the Centre Hospitalier Régional Universitaire of Nancy (CHRU) and supported by the French Ministry of Health (Programme Hospitalier de Recherche Clinique Inter-régional 2013), the Contrat de Plan Etat-Lorraine IT2MP and the Fonds Européen de Développement Régional (FEDER Lorraine), a public grant overseen by the French National Research Agency (ANR) as part of the second Investissements d'Avenir program FIGHT-HF (reference: ANR-15-RHU-0004), and the French Projet investissement d'avenir (PIA) project Lorraine Université d'Excellence (reference ANR-15-IDEX-04-LUE). The STANISLAS study is also supported by the sixth European Union—Framework Program (EU-FP) Network of Excellence Ingenious HyperCare (#LSHM-CT-2006–037093), the seventh EU-FP MEDIA (Européen Cooperation—Theme Health/FP7-HEALTH-2010-single-stage #261409), HOMAGE (Heart Omics in Aging, 7th Framework Program grant #305507), FOCUS-MR (reference: ANR-15-CE14-0032-01), and FIBRO-TARGETS (FP7#602904) projects, and by ERA-CVD EXPERT (reference: ANR-16-ECVD-0002-02).

## Conflict of Interest

SW reports a grant from the International Society of Nephrology, outside of the submitted work. PR received grants and personal fees from Vifor Fresenius Medical Care Renal Pharma, personal fees from Idorsia, personal fees from KBP, Sanofi, NovoNordisk, personal fees from Ablative Solutions, non-financial support from G3P, personal fees from Corvidia, grants, personal fees from Relypsa a Vifor company and Vifor, personal fees from CardioRenal, grants and personal fees from AstraZeneca, grants and personal fees from Bayer, grants and personal fees from CVRx, personal fees from Fresenius, grants and personal fees from Novartis, personal fees from Grunenthal, personal fees from Servier, personal fees from Stealth Peptides, and all outside the submitted work. NG reports personal fees outside of the submitted work from Novartis, Vifor and AstraZeneca. The remaining authors declare that the research was conducted in the absence of any commercial or financial relationships that could be construed as a potential conflict of interest.

## Publisher's Note

All claims expressed in this article are solely those of the authors and do not necessarily represent those of their affiliated organizations, or those of the publisher, the editors and the reviewers. Any product that may be evaluated in this article, or claim that may be made by its manufacturer, is not guaranteed or endorsed by the publisher.

## Abbreviations

CKD: chronic kidney disease
HBI: Healthy Beverage Index
MetS: metabolic syndrome
SSBs: sugar-sweetened beverages
French PNNS: Programme National Nutrition Santé.
