# Supplementary material for: Quality of Beverage Intake and Cardiometabolic and Kidney Outcomes: Insights From the STANISLAS Cohort
Source: Front Nutr. 2022 Jan 7;8:738803. doi: 10.3389/fnut.2021.738803 (PMC8777230; doi:10.3389/fnut.2021.738803)
Supplement: Supplementary file 1 [file Table_1.DOCX]

Supplementary table 1. Beverage consumption in the overall population and according to Healthy Beverage Index quartiles. Mean (SD) are shown for each variable.

|  | **Overall** | **<= 78** | **79-89** | **90-95** | **> 95** | **p** |
| --- | --- | --- | --- | --- | --- | --- |
| N | 1302 | 313 | 335 | 325 | 329 |  |
| Water intake (mL) | 973.3 (530.0) | 671.0 (551.2) | 843.6 (447.0) | 1030.3 (419.0) | 1336.8 (456.1) | <0.001 |
| Coffee intake (mL) | 286.3 (285.4) | 274.9 (275.7) | 262.0 (278.2) | 304.8 (301.8) | 303.8 (284.2) | 0.137 |
| Tea or herbal tea intake (mL) | 197.5 (304.7) | 134.1 (260.1) | 154.2 (276.7) | 236.3 (311.7) | 263.6 (344.1) | <0.001 |
| Low-fat milk intake (mL) | 72.4 (136.5) | 113.9 (211.0) | 76.8 (119.6) | 57.0 (92.1) | 43.6 (76.9) | <0.001 |
| Diet soda intake (mL) | 23.5 (101.2) | 29.3 (123.1) | 29.0 (125.2) | 13.6 (51.9) | 22.1 (86.2) | 0.043 |
| Fruit juice intake (mL) | 61.6 (99.0) | 103.9 (143.0) | 64.4 (96.5) | 47.7 (65.6) | 32.0 (53.8) | <0.001 |
| Alcohol intake (mL) | 132.4 (177.3) | 270.6 (274.0) | 119.3 (122.7) | 76.7 (83.2) | 69.1 (75.4) | <0.001 |
| Alcohol intake (servings) | 0.9 (1.2) | 1.9 (1.9) | 0.9 (1.0) | 0.5 (0.6) | 0.5 (0.5) | <0.001 |
| Whole milk intake (mL) | 6.7 (37.4) | 12.9 (53.4) | 9.9 (43.8) | 3.9 (27.8) | 0.1 (1.7) | <0.001 |
| Soda intake (mL) | 46.9 (145.1) | 138.4 (267.7) | 26.6 (53.3) | 14.9 (31.6) | 12.1 (29.7) | <0.001 |
| Energy intake from beverages (kcal) | 182.3 (161.6) | 361.8 (210.0) | 168.1 (94.6) | 114.6 (72.5) | 92.8 (62.1) | <0.001 |
| Beverage volume (mL) | 1800.5 (681.8) | 1749.1 (851.6) | 1585.8 (607.8) | 1785.2 (560.8) | 2083.2 (580.7) | <0.001 |
| Total energy intake (kcal/d) | 2359.7 (806.1) | 2598.9 (869.3) | 2620.2 (875.7) | 2306.5 (670.2) | 1919.4 (559.4) | <0.001 |
